# Modification and Validation of a Reference Real-Time RT-PCR Method for the Detection of a New African Horse Sickness Virus Variant

**DOI:** 10.3390/microorganisms13122684

**Published:** 2025-11-25

**Authors:** Jorge Morales, María José Ruano, Cristina Tena-Tomás, Antoinette van Schalkwyk, Eleni-Anna Loundras, Marta Valero-Lorenzo, Ana López-Herranz, Marco Romito, Carrie Batten, Rubén Villalba, Montserrat Agüero

**Affiliations:** 1 Laboratorio Central de Veterinaria, Ministry of Agriculture, Fisheries and Food, 28110 Algete, Spain; 2 Tecnologías y Servicios Agrarios, S.A. (TRAGSATEC), 28037 Madrid, Spain; 3 Agricultural Research Council, Onderstepoort Veterinary Institute, Onderstepoort 0110, South Africa; 4 The Pirbright Institute, Woking GU24 0NF, UK

**Keywords:** African horse sickness, AHSV, new strain, rRT-PCR, reference method, validation

## Abstract

African horse sickness (AHS) is a disease affecting equids caused by the AHS virus (AHSV). The World Organisation for Animal Health (WOAH) includes AHS as a notifiable disease and, upon detection within the European Union, immediate control and eradication measures are mandated. Thus, validated diagnostic methods for rapid AHSV detection are essential. The Agüero 2008 and Guthrie 2013 rRT-PCR methods have been widely validated for detection of any AHSV strain and are included as reference rRT-PCRs in the WOAH manual. However, the WOAH Reference Laboratory for AHS in the Republic of South Africa (RSA) reported an AHSV variant undetected by the Agüero 2008 rRT-PCR. Therefore, a set of modified primers and probe, containing degenerate positions to avoid mismatches with the sequence of the new RSA strain, was developed. The modified-Agüero method was validated by the WOAH Reference Laboratories in Spain and the UK, employing a broad collection of AHSV strains and clinical samples as well as a synthetic RNA mimicking the target sequence of the new RSA AHSV variant (AHSV-sRNA-RSA). Comparative assessment of the modified-Agüero versus the WOAH reference rRT-PCRs showed that the modified method exhibited good diagnostic performance and enabled detection of the new RSA AHSV variant nucleic acid.

## 1. Introduction

African horse sickness (AHS) is a viral disease that affects equids. It is caused by the African horse sickness virus (AHSV), an orbivirus from the Sedoreoviridae family, and transmission occurs through the bite of Culicoides, making environmental factors and midge population densities critical in the spread of the disease [1,2,3]. The fact that nine distinct serotypes of AHSV have been identified is significant for diagnostic and vaccination purposes [1,3,4,5,6,7,8,9]. The variety of clinical presentations in AHS adds significant complexity to its management and control. The four forms—horse sickness fever, cardiac, mixed, and pulmonary forms—differ in severity, with the pulmonary form being particularly devastating [3,4]. The high mortality rate associated with the respiratory form, sometimes exceeding 95% in non-vaccinated animals, underscores the critical importance of vaccination in preventing outbreaks and reducing fatalities [1,3,4]. Besides the relevant impact of AHS in equid welfare, it is estimated that African horse sickness results in an annual economic loss of approximately USD 95 million in sub-Saharan Africa, where it is endemic [10].

The geographical spread of AHS outside sub-Saharan Africa highlights the potential for the disease to affect equine populations globally. Historical outbreaks in the Middle East and North Africa and later in Europe and Asia show how quickly the virus can spread, especially when conditions are conducive for the vector to thrive [1,3]. The outbreak caused by AHSV-4 in Spain, Portugal, and Morocco in the late 1980s, and more recently the outbreak in Southeast Asia caused by AHSV-1 [5,6,7,11], further exemplify the global risks posed by the disease to animal health and the equine industry worldwide. The rapid spread of AHS and its high mortality rate in unvaccinated horses emphasise the need for effective surveillance, vaccination programs, and vector control strategies, especially in non-endemic regions, to mitigate the impact of this serious disease [10,12,13].

The World Organisation for Animal Health (WOAH) includes AHS as a notifiable disease, reflecting the global importance of monitoring and controlling the disease [9]. Therefore, AHS must be reported by affected countries, enabling the WOAH to assist in coordination of global response efforts to prevent further spread of the disease [14]. Within the European Union (EU), AHS is considered one of the animal diseases for which immediate control and eradication measures are mandated upon AHSV detection. This is included in the EU’s comprehensive “Animal Health Law”, which sets protocols for disease management, surveillance, and eradication, helping to prevent outbreaks that could impact both equine health and the agricultural economy in the EU [8]. The international legislative frameworks emphasise the importance of vigilance, rapid response, and collaboration to minimise the impact of AHS and protect equine populations worldwide. To meet these goals, the availability of validated, effective, and harmonised diagnostic methods is essential. In this regard, there are several real-time reverse-transcription polymerase chain reaction (rRT-PCR) methods available for AHS serogroup detection [15,16,17,18,19,20] and serotype identification [20,21,22,23]. Specifically, the rRT-PCR methods described by Agüero [15] and Guthrie [19,24] are included as reference rRT-PCRs for AHSV detection in the WOAH manual [25]. Both methods target segment 7 of the viral genome, because it is highly conserved among AHSV strains [12] and the amplicons they generate partially overlap. These rRT-PCRs have allowed the detection of any circulating AHSV strain, regardless of the serotype. Currently, the Agüero 2008 rRT-PCR is widely used by EU National Reference Laboratories (EU-NRLs) for AHSV molecular diagnosis. This method was developed by the EU Reference Laboratory (EURL) for AHS and has been established as a reference rRT-PCR in the field for 16 years, being routinely used for AHSV detection at the EURL. Consequently, this method is widely used amongst EU National Reference Laboratories (EU-NRLs); in fact, 19 out of 27 (70.4%) EU-NRLs participating in the last proficiency test organised by the AHS EURL used the Agüero 2008 as an rRT-PCR method for AHSV serogroup detection (EURL, personal communication).

Recently, Pretoria University (RSA) and the WOAH Reference Laboratory in the RSA at the Agricultural Research Council—Onderstepoort Veterinary Institute (ARC-OVI) reported to the WOAH and EURL for AHS, the Laboratorio Central de Veterinaria (LCV), based in Algete, Madrid (Spain), that the Agüero 2008 rRT-PCR was unable to detect a putatively novel AHSV strain from the Western Cape. The sample submitted to the ARC-OVI was successfully amplified using a hemi-nested assay [26] and typed as serotype 9 [27]. Moreover, it was reported that this strain had been previously detected by the Guthrie 2013 rRT-PCR method.

To further underpin the lack of sensitivity of the Agüero 2008 rRT-PCR to detecting the novel AHSV variant, the WOAH Reference Laboratory in the RSA obtained the partial sequence of segment 7 covering the region targeted by the Agüero 2008 and Guthrie 2013 rRT-PCR methods. Despite being a highly conserved region of the virus genome, some mismatches were identified between the primers and probes of both methods and the new strain target sequence. Consequently, modified primers and probe based on the Agüero 2008 method containing degenerate positions to avoid mismatches were designed, tested in silico, and validated. Given that the new AHSV strain detected in the RSA was not available at the WOAH Reference Laboratory in Spain, two synthetic RNAs containing the target sequence of the Agüero 2008 and Guthrie 2013 rRT-PCRs were designed: one positive control mimicking the target sequence of the reference AHSV strains (AHSV-sRNA-C+), and another with the segment-7 target sequence of the undetected RSA strain (AHSV-sRNA-RSA). These synthetic RNAs were used for the comparative assessment of the modified-Agüero rRT-PCR versus the WOAH manual reference methods, in addition to a wide variety of AHSV strains and clinical samples from the collections kept at the WOAH Reference Laboratories in the UK (The Pirbright Institute) and in Spain (EURL, LCV). In this study we present the optimisation and validation of the modified-Agüero method, which allows the reliable detection of the new RSA AHSV strain, as well as the AHSV collection strains and clinical samples.

## 2. Materials and Methods

### 2.1. Nucleic Acid Extraction

An amount of 200 μL of EDTA blood samples, tissue homogenates, or viral suspensions was subjected to nucleic acid extraction using the BioSprint^®^ 96 DNA Blood Kit (Qiagen, Hilden, Germany). Nucleic acids were eluted in a final volume of 50 μL of nuclease-free water and kept at −80 °C until use.

### 2.2. Primer and Probe Design

The primer and probe sequences of the original Agüero method [15] were modified to target the RSA strain sequence provided by the RSA WOAH Reference Lab (GenBank accession number PV455026). Subsequently, in the reverse primer, AHS-R-A2024 (5′-CTAATGAAAGCGGYGACCGT-3′), a degenerate nucleotide was introduced in the 14th position, whereas in the probe, AHS-R-A2024 (FAM-GCTAGCRGCYTACCACTA-MGB), degenerate nucleotides were included in the 7th and 10th positions. Additionally, these oligonucleotides as well as the ones of the reference RT-PCR methods were aligned with sequences encoding the AHSV VP7 currently available in the GenBank database (n = 227), using ClustalW 1.8 [28] to detect mismatches that may compromise AHSV detection.

### 2.3. Design of the Synthetic AHSV RNAs

A synthetic RNA containing the sequence of the undetected South African strain targeted by the Agüero 2008 and Guthrie 2013 rRT-PCR methods (AHSV-sRNA-RSA) was designed and ordered. Likewise, a synthetic RNA equalling the target sequence shared by most AHSV reference strains (AHSV-sRNA-C+) was designed as a positive control. These nucleic acids, of 120 nucleotides in length each, were synthesised by Metabion International AG (Planegg-Steinkirchen, Germany) (Supplementary Figure S1). Upon arrival, 1 nmol of each dry synthetic RNA pellet was used to prepare specific solutions containing 6.023 × 10^9^ molecules/μL of each synthetic RNA in molecular grade water and kept at −80 °C until further use. Ten-fold serial dilutions of these synthetic RNA solutions were used for the comparative assessment of the diagnostic performance of the modified-Agüero method versus the WOAH reference rRT-PCR methods.

### 2.4. Serogroup-Specific rRT-PCR

To test the performance of the modified primer and probe, rRT-PCR assays were carried out in the same conditions as the original method following the rRT-PCR protocol described in [29]. Briefly, rRT-PCR was performed in a final volume of 20 μL using the commercial kit AgPath-ID^TM^ One-Step RT-PCR Reagents (Applied BioSystems, Whaltman, MA, USA). Samples were classified as positive when a typical amplification curve was obtained and the cycle threshold (Ct) value was lower than or equal to 35 within 40 PCR cycles (Ct ≤ 35), inconclusive when 35 < Ct < 40, and negative when a Ct > 40 or no Ct was obtained.

### 2.5. Viral Strains and Clinical Samples

The nine AHSV reference strains (serotypes 1 to 9) and thirty-four additional isolates from the EURL’s AHSV reference collection were used for the validation of the modified-Agüero rRT-PCR. Further, eleven AHSV strains from The Pirbright Institute presenting mismatches with the Agüero 2008 and Guthrie 2013 primers and/or probe were included in this validation to evaluate the inclusivity of the modified method. To assess the specificity (exclusivity), representative strains of various orbiviruses were employed, including reference strains of bluetongue virus (BTV) corresponding to notifiable serotypes 1 to 24, epizootic haemorrhagic disease virus (EHDV) serotypes 1, 2, 4, 5, 6, 7, and 8, and equine encephalosis virus (EEV) serotype 3. Additionally, viruses known to induce disease in equids, such as West Nile virus lineage 1 and equine herpesvirus serotypes 1 and 4, were incorporated (Supplementary Table S1). All orbivirus strains used in this investigation from the EURL collection were propagated in BHK-21 clone 13 cells (American Type Culture Collection, ATCC-CCL-10TM), Vero cell monolayers (ATCC-CCL81), or KC cells derived from *Culicoides sonorensis* [30]. Control rRT-PCR analyses included uninfected cell lines. Clinical samples of equines were used to assess the diagnostic parameters, which included 30 EDTA blood and 25 tissue samples from the 2015–2017 outbreaks in Kenya, which is an AHS-endemic country, 24 spleen samples from the AHS outbreak in Spain (1987–1990), and 28 EDTA blood samples corresponding to horses from Spain obtained between 2019 and 2022 (AHS-free status country). Tissue samples were collected post-necropsy and preserved by storage at ultra-low temperatures (−80 °C). Viral suspensions were prepared through serial passages in cell cultures (KC, Vero, or BHK-21), followed by clarification via centrifugation at 805× *g* for 10 min, and subsequently stored at −80 °C. EDTA blood samples were maintained at refrigerated conditions between 2 and 8 °C or stored at −80 °C until laboratory analysis.

### 2.6. rRT-PCR Validation Parameters Evaluated

Validation parameters of the modified-Agüero method were mainly assessed by comparison to the reference rRT-PCR method Agüero 2008. Analytical sensitivity was determined using ten-fold serial dilutions of the nine AHSV reference strains (serotypes 1 to 9). Analytical specificity (exclusivity) of the modified rRT-PCR method was evaluated by analysing other orbiviruses, several viruses affecting horses, and the most common cell lines used for AHSV propagation. Analytical specificity (inclusivity) was assessed using the EURL’s AHSV strain collection. Additionally, to further appraise inclusivity, several AHSV strains from The Pirbright Institute were analysed by using the three methods Agüero 2008, Guthrie 2013, and modified-Agüero. The diagnostic performance of the assay was evaluated using clinical samples (EDTA blood and tissue samples) from the AHS outbreaks in Spain in 1987–1990, samples from the 2015–2017 outbreaks in Kenya, and horse blood samples from free-of-AHS areas. Finally, intra-assay repeatability was checked by performing the analytical sensitivity tests in duplicate and calculating the Ct difference between assays.

### 2.7. Statistical Analysis

To evaluate the validation parameters of the modified-Agüero method, statistically significant differences (*p* < 0,05) among sets of Ct values obtained from samples analysed with the Agüero 2008 and the modified-Agüero rRT-PCRs were assessed using the *t*-test with Microsoft Excel software. A one-way ANOVA test followed by a Fisher least significant difference (LSD) post-hoc test was used to compare the diagnostic performance of the three rRT-PCR methods, using XLSTAT (v.2025.1.3) Excel data analysis add-on software [31].

## 3. Results

### 3.1. In Silico Analysis of the Novel South African AHSV Strain Sequence

**Figure 1:**
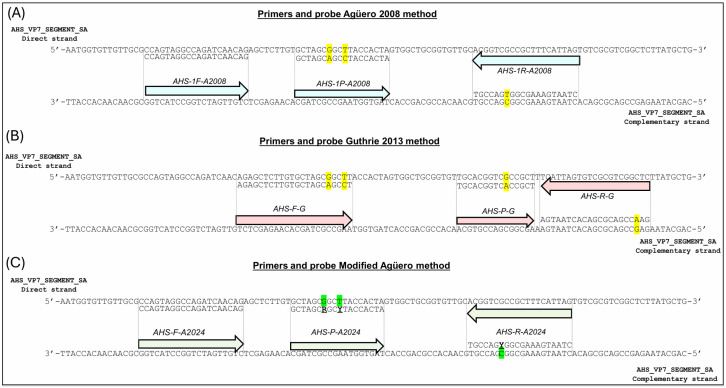
Alignment of the primers/probe described by Agüero (2008), Guthrie (2013), and the modified-Agüero method with segment 7 of the new AHSV strain from the RSA. (**A**) Alignment of the forward (AHS-1F-A2008) and reverse (AHS-1R-A2008) primers and probe (AHS-1P-A2008) of the Agüero 2008 method with the target sequence of the AHSV strain from the RSA. (**B**) Alignment of the forward (AHS-F-G) and reverse (AHS-R-G) primers and probe (AHS-P-G) of the Guthrie 2013 method with the target sequence of the AHSV strain from the RSA. (**C**) Alignment of the forward (AHS-1F-A2024) and reverse (AHS-1R-A2024) primers and probe (AHS-P-A2024) of the modified-Agüero rRT-PCR method with the target sequence of the AHSV strain from the RSA. The sequences are shown as cDNA; the primers and probes are depicted by coloured arrows and located at the annealing site of the cDNA target strand. The sequence of the primers and probes is shown aligned with the opposite strand to facilitate the visualisation of mismatches. Mismatches are highlighted in yellow and modifications of the primer/probe are shown in green.

To detect mismatches that could potentially explain the lack of sensitivity of the Agüero rRT-PCR, an in silico comparison of the primers/probe of the Agüero and Guthrie methods with the sequence of the undetected South African virus strain provided by the ARC-OVI was performed. As shown in Figure 1A, mismatches were detected in the reverse primer (one position, T to C at the 14th nucleotide from the 5′ primer end) and probe (two positions, A to G and C to T at the 7th and 10th nucleotides from the 5′ probe end, respectively) of the Agüero 2008 method. As for the Guthrie primers and probe, two mismatches were found in the forward primer, one in the probe and one in the reverse primer (Figure 1B). Accordingly, modifications were made to generate an optimised reverse primer and probe based on the Agüero 2008 method (Figure 1C).

Subsequently, the primer/probe sequences of the original and modified methods were compared in silico with 227 AHSV sequences deposited in GenBank. There were 110 and 13 AHSV sequences showing at least one mismatch with Agüero 2008 and Guthrie 2013, classified in 14 and 3 different patterns, respectively (Table 1).

### 3.2. Validation of the Modified-Agüero rRT-PCR

To assess exclusivity, other orbiviruses, several viruses affecting horses, and the most common cell lines used to propagate AHSV (Supplementary Table S1) were tested. Similarly to the exclusivity shown by the Agüero 2008 method, these samples were not detected with the modified-Agüero rRT-PCR.

Analytical sensitivity was assessed by testing ten-fold serial dilutions of reference strains from all nine AHSV serotypes using both modified and original methods in parallel, in two technical replicates. All reference strains of the nine viral serotypes were properly detected by the modified method, in the same concentration range as the original method (Table 2). These results show that the modified rRT-PCR exhibits a similar analytical sensitivity as compared to the original method.

Inclusivity was evaluated by analysing viral suspensions from the AHSV EURL’s strain collection, showing that all the strains were properly detected by the modified-Agüero method and similar Ct values were obtained with both methods (mean Ct = 21.33 ± 4.79 SD vs. 21.89 ± 5.31 SD; *p*-value = 0.64; Supplementary Table S2).

To further ensure the inclusivity of the modified-Agüero method, additional strains of the AHSV collection maintained at Pirbright were analysed. To this end, sequences of the modified-Agüero primers/probe were provided to the WOAH Reference Laboratory for AHS in the UK, where analyses of AHSV strains in their virus collection showing mismatches with primers/probes of the reference methods were carried out. The sequences of these AHSV strains correspond to some strain patterns described in Table 1 and some strains that have additional mismatches (Table 3).

As shown in Table 3, the three methods detected the AHSV strains presenting one or two mismatches with the primers or probe. Likewise, viral strains showing one mismatch in any of the primers/probe or one mismatch in a primer plus a mismatch in the other primer or the probe have been properly detected with the three rRT-PCR methods.

Regarding diagnostic performance, similar Ct values were obtained with both methods from convalescent horses’ EDTA blood (Agüero 2008 mean Ct = 28.31 ± 2.34 SD vs. modified-Agüero mean Ct = 27.64 ± 2.44 SD; *p*-value= 0.31) and tissue samples (Agüero 2008 mean Ct = 26.18 ± 4.2 SD vs. modified-Agüero mean Ct = 25.99 ± 4.3 SD; *p*-value= 0.88) obtained during the Kenya outbreaks (Supplementary Table S3). In addition, twenty-four (24) spleen samples from affected horses in Spain (1987–1990 outbreaks) were analysed using both methods (Supplementary Table S4). Again, comparable Ct values were obtained (Agüero 2008 mean Ct = 27.34 ± 4.15 SD vs. modified-Agüero method mean Ct = 26.37 ± 3.41 SD; *p*-value = 0.38). Thus, our data demonstrate that the diagnostic sensitivity of the modified-Agüero rRT-PCR is comparable to that of the Agüero 2008 method in clinical samples (blood and tissues). Overall, from seventy-five (75) analysed positive clinical samples, the agreement in the results was 100% without significant differences in the Ct values obtained. To check diagnostic specificity, twenty-eight (28) EDTA blood samples from horses from an AHS-free country were analysed. As expected, both methods yielded negative results.

To complete the evaluation of the modified-Agüero rRT-PCR validation parameters, intra-assay repeatability was assessed. The observed variation in Ct values between technical replicates was low (mean absolute Ct value difference = 0.9 ± 0.84 SD) (Supplementary Table S5).

### 3.3. Comparative Assessment to Detect the New AHSV Variant from the RSA

To test the ability of the modified-Agüero method to detect the new AHSV variant from the RSA, a comparative assessment with the Agüero 2008 and the Guthrie 2013 methods was performed using synthetic RNAs mimicking the AHSV reference strain (AHSV-sRNA-C+) and the RSA undetected AHSV strain (AHSV-sRNA-RSA) as target sequences. The synthetic RNAs were used to prepare a solution containing 10^9^ molecules/μL in water; then, ten-fold serial dilutions were obtained and 2 μL of each dilution was tested with the three rRT-PCR methods. As expected, the three methods detected the positive control (AHSV-sRNA-C+) (Table 4).

Specifically, the Guthrie 2013 method presented a statistically significant lower mean Ct value to detect this AHSV-sRNA-C+ as compared to the Agüero 2008 rRT-PCR (mean Ct value difference = 5.87 * ± 0.82 SD, *p*-value = 0.012). No statistically significant differences were observed between the Ct values obtained with the modified-Agüero method and the WOAH reference rRT-PCRs (mean Ct value difference with the Guthrie method = 3.37 ± 1.01 SD, *p*-value = 0.141; mean Ct value difference versus the Agüero 2008 method = 2.50 ± 0.50 SD, *p*-value = 0.272).

As shown in Table 5, the AHSV-sRNA-RSA was undetected by the Agüero 2008 rRT-PCR whereas it was positively ascertained by the modified-Agüero and the Guthrie 2013 methods. Interestingly, the observed Ct values using the modified-Agüero method were consistently lower than those obtained by the Guthrie rRT-PCR, independently of the target AHSV-sRNA-RSA dilution (mean Ct value difference = 3.83 ± 0.6 SD, *p*-value = 0.081).

## 4. Discussion

Given the relevance of AHS, effective vigilance, rapid response, and collaboration to reduce its impact and protect equine populations are key to minimising the risks posed by the disease to animal health and the equine industry worldwide [10,12,13]. To meet these goals, the availability of validated and harmonised AHS diagnostic methods is essential. The Agüero 2008 is one of the two rRT-PCR methods for AHSV molecular diagnosis included in the WOAH manual [25], and it is used worldwide. Moreover, EU Regulation 2016/429 of the European Parliament and of the Council, article 94 point 1, states that EURLs shall contribute to the improvement and harmonisation of methods of analysis, tests, or diagnoses to be used by official laboratories designated [8]. Therefore, upon notification by the WOAH Reference Laboratory in the RSA that the Agüero 2008 rRT-PCR was unable to detect a new AHSV strain from the Western Cape, as a EURL and WOAH Reference Laboratory for AHS, we conducted the modification and validation of the Agüero 2008 rRT-PCR method.

The in silico comparison of the primers/probe of the Agüero 2008 and Guthrie 2013 rRT-PCRs with the sequence of the undetected South African virus provided by the RSA WOAH Reference Laboratory revealed mismatches with the reverse primer (one position) and probe (two positions) of the Agüero 2008 method. Since PCR with degenerate primers can be used to detect genetic variants [32], we optimised the sensitivity of the Agüero 2008 method by introducing degenerate nucleotides at the mismatch positions of the reverse primer and probe. These changes allowed better complementarity and annealing than the Agüero 2008 reverse primer and probe with the novel RSA AHSV strain segment-7 target sequence, while maintaining good complementarity with the target sequences of AHSV reference and collection strains.

We demonstrated that the modified assay exhibited similar analytical sensitivity to the original Agüero 2008 method, while analytical specificity (exclusivity) was maintained. A slight variation in the analytical sensitivity between the original and modified methods was observed. In this regard, reproducibility between RT-PCR assays has been stablished in 2.1 Cts at the EURL.The absence of differences in mismatches between the primers and probe of the modified versus the original assay with the target sequences of the AHSV reference strains (Supplementary Figure S2) indicates that the minor differences observed between methods might be due to the intrinsic variation in the RT-PCR assay. Inclusivity was evaluated by using an extensive AHSV reference collection from the EURL and The Pirbright Institute, including AHSV strains showing some mismatches in primers and/or the probe. Moreover, the diagnostic performance results obtained using samples from convalescent horses taken during AHS outbreaks and from negative horses from AHS-free zones showed that the modified-Agüero method presents an equivalent diagnostic specificity and sensitivity as compared to the original Agüero 2008 rRT-PCR. Overall, the data obtained from the comparative analyses demonstrated that the modified-Agüero 2008 rRT-PCR shows similar analytical and diagnostic performance as compared with the original Agüero 2008 method. Also, the observed variation in Ct values between replicates was low, showing good repeatability of the assay.

Although the RSA WOAH Reference Laboratory shared the sequence of the new AHSV RSA strain targeted by the Agüero 2008 and Guthrie 2013 rRT-PCRs, the shipment of this virus to the AHS EURL was not possible. Since synthetic RNAs have been successfully used as spike-in controls in RNA-seq experiments [33], we designed a synthetic RNA of 120 nucleotides containing the amplicon generated by the Agüero 2008 method (AHSV-sRNA-RSA). This synthetic nucleic acid was used to carry out a comparative assessment of the diagnostic performance of the modified-Aguero method versus the WOAH reference rRT-PCR methods.

The results of the comparative assessment of the modified-Agüero versus the WOAH manual reference methods showed that all three rRT-PCRs detected the positive control AHSV-sRNA-C+. In this regard, our data show a lower Ct value of the Guthrie 2013 method for this positive control as compared to the Agüero 2008 rRT-PCR, and a similar sensitivity to the modified-Agüero rRT-PCR. With respect to the AHSV-sRNA-RSA, it was not detected by the Agüero 2008 rRT-PCR but was positively identified by the modified-Agüero and the Guthrie 2013 methods. The explanation for why the original Agüero 2008 method with a total of three mismatches (one in the reverse primer and two in the probe) was unable to detect the relevant RSA strain, whereas the Guthrie method with four mismatches (two in the forward primer, one in the probe, and one in the reverse primer) was able to detect the RSA strain, remains hitherto elusive. A plausible explanation is that the effects of mismatches in the probe binding region, could be more pronounced for a broad range of characteristics of real-time PCR amplification curves than mismatches in the primer [34]. Total complementarity of the probe with the target sequence allows correct hybridisation and stability between the probe and the complementary strand during the elongation step, which is required for the 5′ exonuclease activity of the polymerase to degrade the probe and release the fluorochrome group from the 5′ position [35,36]. In this regard, our data indicate that the two mismatches of the Agüero 2008 probe with the RSA variant target sequence might affect the melting temperature of the probe enough to prevent its stable hybridisation during the elongation step and, therefore, not be degraded by the polymerase and not generate an increase in fluorescence. Therefore, the original Agüero 2008 method could be more impaired in detecting the new RSA AHSV strain than the modified-Agüero and the Guthrie methods. Interestingly, the observed Ct values obtained using the modified-Agüero method were lower than those attained with the Guthrie rRT-PCR at any target AHSV-sRNA-RSA dilution, suggesting a higher sensitivity of the modified-Agüero versus the Guthrie method to detect this strain. This can be due to the better complementarity of the optimised primer and probe of the modified-Agüero method with the new RSA AHSV strain target sequence.

To further ensure that the modified-Agüero method was able to detect a wide range of reference AHSV strains, particularly those including mismatches with the primers and/or probes of any of the three methods, the diagnostic performance of the modified-Agüero rRT-PCR was evaluated and compared with that of the WOAH reference rRT-PCRs using several strains of AHSV collection from Pirbright’s orbivirus reference collection. These assays were part of the inclusivity assessment of the modified-Agüero method and were only performed on virus strains that had mismatches with the primers and/or probe of that specific rRT-PCR, which included strains from Senegal [37] and from AHS outbreaks in Ethiopia in 2010 [38] and Thailand in 2020 [5]. These data show that the three methods detect the AHSV strains from the Pirbright orbivirus reference collection despite the presence of one or two mismatches in the primer and/or probe and further reinforce the adequate diagnostic performance of the modified-Agüero method.

It is noteworthy that, even though segment 7 is highly conserved in AHSV [12,39], almost half of the AHSV strains deposited in GenBank from 2009 to 2023 have at least one mismatch with respect to the primers and/or probe sequences of the Agüero 2008 method, and 13 AHSV strains have mismatches with respect to the primers and/or probe of the Guthrie method. However, no detection failures with the reference methods have been reported to date. Among these strains with mismatches, the ones deposited in GenBank and those from Pirbright’s collection, none present two mismatches in the probe. Nevertheless, the new AHSV strain from the RSA not detected by the Agüero 2008 method is the only one that presents two mismatches in the probe, which, in agreement with Süss and cols., suggests that this might be critical for the rRT-PCR’s sensitivity.

The two reference methods have proven to be the most sensitive and specific of all published methods, as described in the WOAH manual [25]. However, both methods target the same region of segment 7, and even the amplicons generated by both methods have overlapping regions. Considering that the occurrence of AHSV strains with mutations in the sequence of this area seems higher than expected for such a conserved segment, it seems advisable to have available additional rRT-PCR methods with similar sensitivity targeting other segments of the AHSV genome, which could be used in case of suspected detection failure. In this regard, there are two rRT-PCR methods targeting segments 1 and 8 currently in development with promising results (Hofmann et al., personal communication).

In conclusion, this study shows that the modified-Agüero rRT-PCR allows the reliable detection of a wide collection of AHSV strains and clinical samples and a synthetic RNA mimicking the new AHSV strain from the RSA, the only one that has been reported so far as undetected by the original Agüero 2008 method. Therefore, we will propose substituting the Agüero 2008 AHS rRT-PCR with the modified-Agüero method in the WOAH manual. Finally, we recommend to AHS EU-NRLs and official diagnostic laboratories worldwide the implementation of the modified-Agüero method described here. This work highlights the relevance of the collaboration between WOAH Reference Laboratories to provide effective and robust diagnostic tools, updated to identify novel viral strains.

## Figures and Tables

**Table 1:** AHSV segment-7 sequences from GenBank (2009–2023) showing mismatches with primers and probes.

**Pattern**	**Number of Sequences**	**GenBank Accession Number (AN)**	**Description of Mismatches with the Agüero 2008 Primers and Probe** **Nucleotide Position Change from 5′**		
			**Forward**	**Probe**	**Reverse**
A1	17	KT715637.1 KT715607.1 KT030586.1 KT030616.1 KF860002.1 HM035402.1 HM035399.1 HM035396.1 KP939763.1 KY471520.1 KY471519.1 KP939655.1 KP939759.1 KP939758.1 KP939760.1 KP939761.1 KP939459.1	3-A>G		
A2	1	KT030396.1	11-A>G		
A3	2	HM035370.1 HM035365.1	12-G>A		
A4	20	KT030666.1 KF860042.1 KP009697.1 HM035403.1 HM035376.1 KP940201.1 HM035373.1 KP940190.1 KP940079.1 KP940078.1 KP940076.1 KP940075.1 KP940074.1 KP940073.1 KP940072.1 KP940071.1 KP939886.1 KP939879.1 KP939402.1 U90337.1	15-C>T		
A5	20	MT586218.1 KT030526.1 KT007174.1 KT187063.1 KT187033.1 KT030476.1 KT030466.1 KP009727.1 KP009687.1 KP009627.1 HM035392.1 HM035382.1 HM035378.1 HM035374.1 HM035364.1 HM035361.1 MT711964.1 KP939647.1 KP939537.1 KP939534.1	18-C>T		
A6	1	KP940080.1	15-C>T 6-A>T		
A7	14	KT030416.1 KT030556.1 KT030536.1 HM035389.1 HM035388.1 HM035383.1 HM035381.1 HM035377.1 HM035371.1 HM035363.1 KP940196.1 KP939976.1 KP939971.1 HM035393.1	18-C>T 3-A>G		
A8	18	HM035397.1 HM035390.1 HM035386.1 HM035385.1 HM035384.1 HM035380.1 HM035379.1 HM035368.1 HM035367.1 KP939975.1 KP939970.1 KP939762.1 KP939878.1 KP939882.1 KP939973.1 KP939888.1 KP939887.1 KP939884.1	9-C>T		
A9	1	KP939454.1	9-C>T		8-A>G
A10	1	KP939655.1	3-A>G	10-C>T *	
A11	9	KP940199.1 KP940193.1 KP939657.1 KP939652.1 KP939650.1 KP939648.1 KP939536.1 KP939535.1 KP939456.1		10-C>T *	
A12	2	KP939767.1 KP939454.1			8-A>G
A13	3	KT715617.1 KT030636.1 KP939881.1			4-A>G
A14	1	KT030546.1			15-G>A
**Pattern**	**Number of Sequences**	**GenBank Accession Number (AN)**	**Description of Mismatches with the Guthrie Method Primers and Probes** **Nucleotide Position Change from 5′**		
			**Forward**	**Probe**	**Reverse**
G1	10	KP939650.1 KP939648.1 KP939456.1 KP939535.1 KP939536.1 KP939652.1 KP939657.1 KP 939655.1 KP940193.1 KP940199.1	21-C>T		
G2	2	KT715617.1 KP939881.1			18-A>G
G3	1	KT939762.1			5-C>T

* This mismatch was corrected by modifying the probe in the modified-Agüero method.

**Table 2:** Analytical sensitivity of the modified-Agüero rRT-PCR compared with the Agüero 2008 method, using ten-fold serial dilutions of the nine AHSV reference strains (serotypes 1 to 9).

Log_10_ Dilution (TCID_50_/_mL_)	Agüero 2008 (Ct)	Modified-Agüero (Ct)	Log_10_ Dilution (TCID_50_/_mL_)	Agüero 2008 (Ct)	Modified-Agüero (Ct)
**AHSV Serotype 1**			**AHSV Serotype 2**		
−2 (10^4.6^)	23.13/22.75	20.17/19.06	−2 (10^4.5^)	22.73/23.65	20.43/21.41
−3 (10^3.6^)	26.53/26.91	23.13/23.73	−3 (10^3.5^)	26.81/26.36	25.17/25.36
−4 (10^2.6^)	29.96/30.06	28.48/29.30	−4 (10^2.5^)	30.50/30.58	27.97/28.91
−5 (10^1.6^)	34.53/34.12	34.77/31.12	−5 (10^1.5^)	33.40/34.95	32.17/32.98
−6 (10^0.6^)	35.53/neg	37.62/37.74	−6 (10^0.5^)	neg/37.02	35.82/36.93
−7 (10^0.06^)	neg/neg	37.10/neg	−7(10^0.05^)	neg/neg	36.92/neg
−8 (10^0.006^)	neg/neg	neg/neg	−8(10^0.005^)	neg/neg	neg/neg
**AHSV Serotype 3**			**AHSV Serotype 4**		
−2 (10^3.6^)	24.49/23.97 23.97	22.86/21.96 21.96	−2 (10^5.2^)	24.10/23.44	22.21/21.64
−3 (10^2.6^)	27.81/26.82	26.17/26.19	−3 (10^4.2^)	26.80/26.86	25.51/25.15
−4 (10^1.6^)	30.59/30.48	30.10/30.02	−4 (10^3.2^)	30.85/30.51	29.74/28.61
−5 (10^0.6^)	34.64/32.25	32.89/33.14	−5 (10^2.2^)	33.84/34.11	32.38/32.97
−6 (10^0.06^)	neg/neg	37.53/36.32	−6 (10^1.2^)	36.22/neg	36.38/36.95
−7 (10^0.006^)	neg/neg	neg/neg	−7 (10^0.2^)	neg/neg	neg/neg
−8(10^0.0006^)	neg/neg	neg/neg	−8 (10^0.02^)	neg/neg	neg/neg
**AHSV Serotype 5**			**AHSV Serotype 6**		
−2 (10^3.9^)	23.03/23.46	18.49/20.94	−2 (10^5.1^)	23.69/23.38	21.43/20.59
−3 (10^2.9^)	25.80/26.10	27.61/24.09	−3 (10^4.1^)	26.46/26.57	25.52/24.50
−4 (10^1.9^)	30.73/29.33	28.07/28.04	−4 (10^3.1^)	30.18/30.62	30.13/29.43
−5 (10^0.9^)	34.10/35.19	32.56/32.93	−5 (10^2.1^)	34.01/34.21	33.13/33.40
−6 (10^0.09^)	neg/37.46	37.32/37.02	−6 (10^1.1^)	37.26/35.97	36.95/36.25
−7 (10^0.009^)	neg/neg	neg/neg	−7 (10^0.1^)	37.41/neg	neg/neg
−8 (10^0.0009^)	neg/neg	neg/neg	−8 (10^0.01^)	neg/neg	neg/neg
**AHSV Serotype 7**			**AHSV Serotype 8**		
−2 (10^5.1^)	24.80/25.39 25.39	26.07/24.79 24.79	−2 (10^4.6^)	25.04/24.86	23.90/23.75
−3 (10^4.1^)	28.25/28.23	28.16/28.09	−3 (10^3.6^)	28.30/27.87	27.38/27.02
−4 (10^3.1^)	31.14/31.84	31.72/30.79	−4 (10^2.6^)	33.56/32.74	32.18/31.92
−5 (10^2.1^)	36.89/36.39	36.48/35.09	−5 (10^1.6^)	35.54/36.13	34.01/35.27
−6 (10^1.1^)	neg/neg	neg/38.62	−6 (10^0.6^)	neg/neg	38.32/neg
−7 (10^0.1^)	neg/neg	neg/neg	−7 (10^0.06^)	neg/neg	neg/neg
−8 (10^0.01^)	neg/neg	neg/neg	−8 (10^0.006^)	neg/neg	neg/neg
**AHSV Serotype 9**					
−2 (10^5.1^)	24.95/24.87	22.51/23.51			
−3 (10^4.1^)	28.03/27.06	26.35/25.67			
−4 (10^3.1^)	30.72/31.68	29.77/31.30			
−5 (10^2.1^)	34.58/35.77	32.07/34.89			
−6 (10^1.1^)	neg/neg	38.47/37.78			
−7 (10^0.1^)	neg/neg	neg/neg			
−8(10^0.01^)	neg/neg	neg/neg			

TCID_50_, tissue culture infectious dose 50%; AHSV, African horse sickness virus; Ct, cycle threshold; neg, negative sample. Two replicates per dilution are shown.

**Table 3:** Comparative assessment of the Agüero, modified-Agüero, and Guthrie 2013 methods using the TPI AHSV collection strains.

Virus Strain	Agüero 2008		Modified-Agüero		Guthrie 2013	
	Mismatches *	Ct	Mismatches *	Ct	Mismatches *	Ct
ETH 2010/18	Pattern A1	22.54	Pattern A1	22.45	No mismatches	Nd
SEN 1998/01	Pattern A3 plus R 7 (A>G)	35.33	Pattern A3 plus R 7 (A>G)	35.05	R 21 (A>G)	31.86
ETH 2010/08	Pattern A4 plus R 4 (A>G)	27.20	Pattern A4 plus R 4 (A>G)	27.07	Pattern G2	22.49
THA 2020/01	Pattern A5	24.86	Pattern A5	25.11	No mismatches	Nd
ETH 2010/13	Pattern A11	31.19	No mismatches	30.24	Pattern G1	28.53
ETH 2019/02	Pattern A11 plus R 14 (T>C)	25.21	No mismatches	24.24	Pattern G1 plus P 10 (A>G)	23.47
ETH 2019/03		24.89		23.71		22.88
ETH 2019/07		27.84		26.71		25.80
ETH 2019/01	No mismatches	Nd	No mismatches	23.35	Pattern G3	20.61
ETH 2019/04		Nd		23.81		21.00
ETH 2019/05		Nd		23.79		21.17
ETH 2019/06		Nd		24.97		22.30

* Described in relation to patterns included in Table 1, the position of mismatches from 5′ in primer R (R), or probe (P) is indicated; Ct, cycle threshold; Nd: not done (modified-Agüero and Guthrie rRT-PCRs were not performed in strains with no mismatches); ETH, Ethiopia; THA, Thailand; SEN, Senegal. Agüero 2008 and Guthrie 2013 assays were only performed on strains that had mismatches with the primers and/or probe of that specific rRT-PCR.

**Table 4:** Comparative assessment of the Agüero 2008, modified-Agüero, and Guthrie 2013 rRT-PCRs using the synthetic RNA containing the segment-7 target sequence of the AHSV reference strain (AHSV-sRNA-C+).

Reference RNA (AHSV-sRNA-C+)		Agüero 2008 (Ct Value)			Mod-Agüero (Ct Value)			Guthrie 2013 (Ct Value)			Mean Ct Value Absolute Difference		
Log_10_ Dilution	Mol/μL	R.1	R.2	R.3	R.1	R.2	R.3	R.1	R.2	R.3	Agüero 2008 vs. Guthrie	Agüero 2008 vs. Mod-Agüero	Mod-Agüero vs. Guthrie
10^−1^	6.023 × 10^8^	14.58	14.68	15.21	12.17	12.38	13.3	7.96	8.29	8.34	6.63	2.21	4.42
10^−2^	6.023 × 10^7^	17.97	18.05	17.92	15.87	15.43	15.99	11.78	11.07	11.98	6.37	2.22	4.15
10^−3^	6.023 × 10^6^	21.7	21.03	21.23	18.16	19.4	18.9	15.47	17.38	15.07	5.35	2.5	2.85
10^−4^	6.023 × 10^5^	24.95	24.95	24.85	22.56	22.49	22.57	18.49	18.16	18.34	6.59	2.38	4.21
10^−5^	6.023 × 10^4^	29.36	30.04	26.52	26.6	23.17	25.7	23.5	22.8	22.5	5.71	3.48	2.22
10^−6^	6.023 × 10^3^	33.37	33.17	34.72	31.54	32.45	30.63	28.88	29.67	29.02	4.56	2.21	2.35
**Mean Ct value difference (all dilutions) ± SD**											5.87 * ± 0.82	2.50 ± 0.50	3.37 ± 1.01

* Statistically significant values (*p* < 0.05). Mol/µL, molecules per microlitre; R, intra-assay replicate; SD, standard deviation.

**Table 5:** Comparative assessment of the Agüero 2008, modified-Agüero, and Guthrie 2013 rRT-PCRs using the synthetic RNA containing the segment-7 target sequence of the South African undetected strain (AHSV-sRNA-RSA).

RSA Undetected Strain (AHSV-sRNA-RSA)		Agüero 2008 Ct Value			Modified-Agüero Ct Value			Guthrie 2013 Ct Value			Ct Absolute Difference Mod-Agüero vs. Guthrie
Log_10_ Dilution	Mol/μL	R.1	R.2	R.3	R.1	R.2	R.3	R.1	R.2	R.3	
10^−1^	6.023 × 10^8^	-	-	-	10.29	10.59	10.30	13.85	14.18	14.11	3.65
10^−2^	6.023 × 10^7^	-	-	-	13.80	13.44	13.78	17.83	17.88	17.72	4.14
10^−3^	6.023 × 10^6^	-	-	-	17.16	16.84	16.69	20.68	20.65	21.34	3.99
10^−4^	6.023 × 10^5^	-	-	-	21.47	20.44	21.61	24.07	25.34	23.53	3.14
10^−5^	6.023 × 10^4^	-	-	-	22.03	24.54	23.73	28.73	27.49	28.34	4.75
10^−6^	6.023 × 10^3^	-	-	-	29.40	28.53	28.99	30.57	33.77	32.38	3.27
**Mean Ct value difference (all dilutions) ± SD**											3.83 ± 0.6

Mol/µL, molecules per microlitre; R, intra-assay replicate; SD, standard deviation.

## Data Availability

The original contributions presented in this study are included in the article/Supplementary Materials. Further inquiries can be directed to the corresponding author.

## Supplementary Materials

The following supporting information can be downloaded at https://www.mdpi.com/article/10.3390/microorganisms13122684/s1: Figure S1: Synthetic RNA sequences and features; Figure S2: Alignment of the segment-7 target sequences of the 9 AHSV serotype reference strains; Table S1: Details of strains and cell lines included to check exclusivity; Table S2: Diagnostic sensitivity of the modified-Agüero method in viral suspensions from the AHSV strain collection maintained at the LCV; Table S3: Diagnostic performance of the modified-Agüero method in EDTA blood and tissue samples from convalescent horses obtained during the Kenya outbreak; Table S4: Diagnostic performance of the modified-Agüero method in blood EDTA samples from affected horses in Spain during the (1989–1990) outbreak; Table S5: Intra-assay repeatability of the modified-Agüero rRT-PCR method.

## Abbreviations

The following abbreviations are used in this manuscript:

A	Adenine
AHS	African horse sickness
AHSV	African horse sickness virus
AHSV-1	African horse sickness virus serotype 1
AHSV-4	African horse sickness virus serotype 4
AHSV-sRNA-C+	RNA mimicking the target sequence of the reference AHSV strain
AHSV-sRNA-RSA	Synthetic RNA mimicking the target sequence of the new RSA AHSV variant
ANOVA	Analysis of variance
ARC-OVI	Agricultural Research Council—Onderstepoort Veterinary Institute
ATCC	American Type Culture Collection
BHK-21	Baby hamster kidney fibroblast cell line
BTV	Bluetongue virus
C	Cytosine
Ct	Cycle threshold
DNA	Deoxyribonucleic acid
EDTA	Ethylenediaminetetraacetic acid
EEV	Equine encephalosis virus
EHDV	Epizootic haemorrhagic disease virus
EU	European Union
EU-NRL	European Union National Reference Laboratory
EURL	European Union Reference Laboratory
G	Guanine
KC	Culicoides sonorensis cell line
LCV	Laboratorio Central de Veterinaria
LSD	Least significant difference
Mol/µl	Molecules per microlitre
R	Replicate
RNA	Ribonucleic acid
rRT-PCR	Real-time reverse transcriptase polymerase chain reaction
RSA	Republic of South Africa
T	Thymine
TCID_50_	Tissue culture infectious dose 50%
UK	United Kingdom
USD	United States dollars
WOAH	World Organisation for Animal Health
