# Correction: Morales et al. Modification and Validation of a Reference Real-Time RT-PCR Method for the Detection of a New African Horse Sickness Virus Variant. *Microorganisms* 2025, *13*, 2684

**DOI:** 10.3390/microorganisms14040880

**Published:** 2026-04-14

**Authors:** Jorge Morales, María José Ruano, Cristina Tena-Tomás, Antoinette van Schalkwyk, Eleni-Anna Loundras, Marta Valero-Lorenzo, Ana López-Herranz, Marco Romito, Carrie Batten, Rubén Villalba, Montserrat Agüero

**Affiliations:** 1 Laboratorio Central de Veterinaria, Ministry of Agriculture, Fisheries and Food, 28110 Algete, Spain; jmbello@mapa.es (J.M.); mruanor@mapa.es (M.J.R.); mvalero@mapa.es (M.V.-L.); alherranz@mapa.es (A.L.-H.); rvillalba@mapa.es (R.V.); 2 Tecnologías y Servicios Agrarios, S.A. (TRAGSATEC), 28037 Madrid, Spain; at_algete9@mapa.es; 3 Agricultural Research Council, Onderstepoort Veterinary Institute, Onderstepoort 0110, South Africa; vanschalkwyka1@arc.agric.za (A.v.S.); romitom@arc.agric.za (M.R.); 4 The Pirbright Institute, Woking GU24 0NF, UK; eleni-anna.loundras@pirbright.ac.uk (E.-A.L.); carrie.batten@pirbright.ac.uk (C.B.)

In the original publication [1], there was a mistake in Figure 1 as published. The sequence of the forward primer has not changed; it is the same as the one in the original 2008 Agüero method and reads 5′-CCAGTAGGCCAGATCAACAG-3′. However, the first “C” was missing in Figure 1C. The corrected Figure 1 appears below.

The authors state that the scientific conclusions are unaffected. This correction was approved by the Academic Editor. The original publication has also been updated.

## Figures and Tables

**Figure 1:**
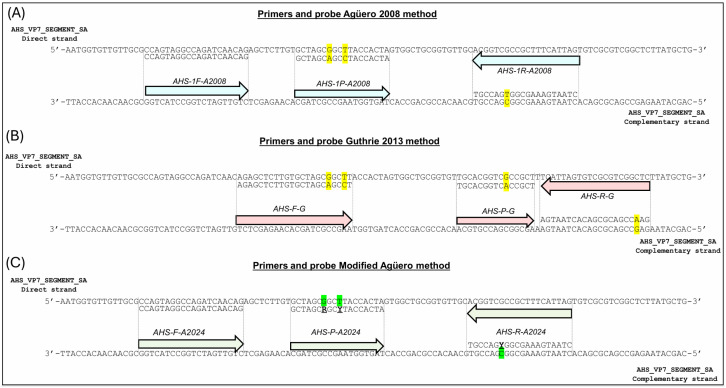
Alignment of the primers/probe described by Agüero (2008), Guthrie (2013), and the modified-Agüero method with segment 7 of the new AHSV strain from the RSA. (**A**) Alignment of the forward (AHS-1F-A2008) and reverse (AHS-1R-A2008) primers and probe (AHS-1P-A2008) of the Agüero 2008 method with the target sequence of the AHSV strain from the RSA. (**B**) Alignment of the forward (AHS-F-G) and reverse (AHS-R-G) primers and probe (AHS-P-G) of the Guthrie 2013 method with the target sequence of the AHSV strain from the RSA. (**C**) Alignment of the forward (AHS-1F-A2024) and reverse (AHS-1R-A2024) primers and probe (AHS-P-A2024) of the modified-Agüero rRT-PCR method with the target sequence of the AHSV strain from the RSA. The sequences are shown as cDNA; the primers and probes are depicted by coloured arrows and located at the annealing site of the cDNA target strand. The sequence of the primers and probes is shown aligned with the opposite strand to facilitate the visualisation of mismatches. Mismatches are highlighted in yellow and modifications of the primer/probe are shown in green.
