# Supplementary material for: Modification and Validation of a Reference Real-Time RT-PCR Method for the Detection of a New African Horse Sickness Virus Variant
Source: Microorganisms. 2025 Nov 25;13(12):2684. doi: 10.3390/microorganisms13122684 (PMC12735099; doi:10.3390/microorganisms13122684)
Supplement: Supplementary file 1 [file microorganisms-13-02684-s001.zip › Supplementary tables.pdf]

Supplementary tables.

Supplementary Table 1. Details of strains and cell lines included to check exclusivity

Virus / Cell line	Strain (Source)
Bluetongue virus (All 24 notified serotypes)	TPI, UK
Equine Encephalosis virus (EEV3 GAM 2009/06)	TPI, UK
Epizootic hemorrhagic disease virus (EHDV1 15.02 [5374])	TPI, UK
Equine rhinopneumonitis type 1	LCV, Spain
Equine rhinopneumonitis type 4	LCV, Spain
West Nile virus (Lineage 1 Egypt 101	Institut Pasteur, France
Usutu virus	LCV, Spain
Equine Eastern Encephalomyelitis	Anses, France
Western Encephalomyelitis	Anses, France
Venezuelan encephalomyelitis	Anses, France
Uninfected Vero cells suspension	ATCC
Uninfected BHK cells suspension	ATCC
Uninfiected KC cells suspension	ATCC

ATCC, American Type Culture Collection; TPI, The Pirbright Institute; LCV, Laboratorio Central de Veterinaria; ANSES, (*Agence nationale de sécurité sanitaire de l'alimentation, de l'environnement et du travail)*; BTV, Bluetongue virus; EHDV, Epizootic haemorrhagic disease virus; Neg, negative; POS, positive (Ct value)

Supplementary Table 2. Diagnostic sensitivity of the modified-Agüero method in viral suspensions from the AHSV strain collection maintained at the LCV

Strain (source)	Agüero 2008 rRT-PCR		Modified-Agüero rRT-PCR	
	Result	Ct	Result	Ct
SEN2007 st2 (TPI)	POS	18.44	POS	18.53
SEN2007 st7 (TPI)	POS	18.18	POS	18.08
KEN2006 st9 (TPI)	POS	18.88	POS	19.04
KEN2007 st4 (TPI)	POS	21.00	POS	21.98
GHA2010 st2 (TPI)	POS	31.51	POS	35.32
ETH2010 st6 (TPI)	POS	19.19	POS	19.34
ETH2010 st2 (TPI)	POS	24.37	POS	23.98
ETH2010 st4 (TPI)	POS	32.68	POS	31.90
ETH2010 st8 (TPI)	POS	23.64	POS	22.08
ETH2010 st9 (TPI)	POS	24.64	POS	22.93
KEN st1 (CVRL)	POS	21.40	POS	20.99
KEN st4 (CVRL)	POS	19.65	POS	19.92
KEN st5 (CVRL)	POS	16.87	POS	17.19
KEN st7 (CVRL)	POS	17.16	POS	17.30
KEN st8 (CVRL)	POS	20.53	POS	20.37
KEN st9 (CVRL)	POS	26.83	POS	26.72
KEN2015 st2 (CVRL)*	POS	18.29	POS	17.81
KEN2013 st4 (CVRL)	POS	20.29	POS	20.03
KEN2015 st7 (CVRL)*	POS	18.60	POS	18.43
KEN2016 st4 (CVRL)*	POS	22.38	POS	23.00
KEN2017 st5 (CVRL)*	POS	19.48	POS	19.66
SPA1988 st4 (LCV)	POS	16.25	POS	15.54
SPA1989 st4 (LCV)	POS	18.45	POS	28.11
SPA1990 st4 (LCV)	POS	21.84	POS	21.26
AHSV1 LAV (OBP)*	POS	14.40	POS	12.20
AHSV2 LAV (OBP)*	POS	17.58	POS	18.39
AHSV3 LAV (OBP)*	POS	17.73	POS	19.12
AHSV4 LAV (OBP)*	POS	31.30	POS	30.91
AHSV6 LAV (OBP)*	POS	26.69	POS	32.31
AHSV7 LAV (OBP)*	POS	18.17	POS	18.70
AHSV8 LAV (OBP)*	POS	17.74	POS	20.57
THA2020 st1 (NIAH)*	POS	31.78	POS	31.54
SPA1988 st4 (LCV O65)	POS	19.78	POS	19.67
SPA1987 st4 (LCV O66)	POS	19.49	POS	21.46
	**Mean Ct**	**21.33**	**Mean Ct**	**21.89**
	**SD**	**4.79**	**SD**	**5.31**
			**Mean Ct absolute difference**	**0,56**
			***p*-value**	**0.142**

AHSV, African Horse Sicknes Virus; st, serotype; POS, positive sample; Ct, cycle threshold; SD, standard deviation; SEN, Senegal; GHA, Ghana; KEN, Kenya; SPA, Spain; R., Sample registry number at the LCV; (OBP), Onderstepoort Biological Products soc ltd. (South Africa); TPI: The Pirbright Institute (UK); CVRL: Central Veterinary Research Laboratory (Dubai); LCV: Laboratorio Central de Veterinaria (Spain); NIAH: National Institute of Animal Health (Thailand); B: BHK; V: Vero; *Virus isolated in the LCV from clinical samples received from this source.

Supplementary Table 3. Diagnostic performance of the modified-Agüero method in EDTA-blood and tissue samples from convalescent horses obtained during the Kenya outbreak

Identification	Type	Serotype	Agüero 2008		Modified-Agüero	
			Result	Ct	Result	Ct
2441/15 17	B	4	POS	30.68	POS	28.34
2441/15 19	B	2	POS	26.92	POS	26.2
2441/15 20	B	5	POS	31.37	POS	31.13
2441/15 13	B	5	POS	24.89	POS	23.48
2782/15 1	B	2	POS	29.89	POS	28.95
2782/15 2	B	2	POS	32.52	POS	33.17
2782/15 3	B	2	POS	24.97	POS	25.75
56/16 2	B	4	POS	26.97	POS	25.66
256/16 3	B	9	POS	26.88	POS	26.85
256/16 4	B	7	POS	27.26	POS	26.04
377/16 2	B	4	POS	29.05	POS	28.88
377/16 3	B	4	POS	26.39	POS	25.52
841/16 8	B	4	POS	28.04	POS	27.14
841/16 9	B	7	POS	25.26	POS	24.52
841/16 10	B	2	POS	28.1	POS	28.09
1766/16 2	B	2	POS	29.4	POS	28.31
1766/16 3	B	2	POS	29.15	POS	28.26
1766/16 4	B	2	POS	29.12	POS	27.94
1766/16 5	B	4	POS	29.52	POS	28.44
2459/16 2	B	4	POS	32.29	POS	30.74
2459/16 3	B	9	POS	30.03	POS	27.59
2459/16 4	B	4	POS	28.09	POS	26.95
2459/16 5	B	3	POS	30.65	POS	30.25
2772/16 1	B	9	POS	25.19	POS	22.34
1083/17 1	B	5	POS	23.95	POS	27.2
1838/17 1	B	5	POS	29.57	POS	30.87
2441/15 (11)	S	5	POS	25.39	POS	27.52
2441/15 (16)	S	4	POS	28.71	POS	28.34
2782/15 (4)	Li	2	POS	28.95	POS	28.22
2782/15 (5)	S	2	POS	27.33	POS	26.2
2782/15 (6)	Lu	2	POS	20	POS	18.65
256/16 (5)	Lu	4	POS	33.38	POS	31.98
256/16 (6)	H	4	POS	32.54	POS	21.8
256/16 (7)	S	4	POS	24.7	POS	24.08
256/16 (8)	Li	4	POS	25.66	POS	23.77
256/16 (9)	Lu	9	POS	31.56	POS	30.77
256/16 (10)	H	9	POS	22.4	POS	20.88
256/16 (11)	S	9	POS	27.16	POS	24.78
256/16 (12)	Li	9	POS	26.35	POS	23.8
1766/16 (8)	H	4	POS	29.79	POS	28.94
1766/16 (9)	Lu	4	POS	33.67	POS	32.61
2459/16 (6)	Lu	7	POS	24.49	POS	22.7
2459/16 (9)	Li	7	POS	26.48	POS	24.49
2772/16 (3)	H	4	POS	26.85	POS	26.29
2772/16 (4)	S	4	POS	26.95	POS	33.95
2772/16 (5)	S	4	POS	26.53	POS	30.81
1083/17 (3)	Lu	5	POS	21.53	POS	23.65
1083/17 (5)	S	5	POS	23.76	POS	26.26
1083/17 (7)	Lu	5	POS	17.46	POS	18.52
1083/17 (8)	H	5	POS	23.21	POS	30.15
1083/17 (9)	Li	5	POS	19.55	POS	20.7
**Statistical analysis of blood samples**			**Mean Ct**	**28.31**	**Mean Ct**	**27.64**
			**SD**	**2.34**	**SD**	**2.44**
			**Mean Ct absolute difference**			**0.67**
			***p*-value**			**0.31**
**Statistical analysis of tissue samples**			**Mean**	**26.18**	**Mean**	**25.99**
			**SD**	**4.20**	**SD**	**4.3**
			**Mean Ct absolute difference**			**0.18**
			***p*-value**			**0.88**

POS, positive sample; Ct, cycle threshold; SD, standard deviation; B, blood-EDTA; S, spleen; Li, liver; Lu, Lung; H, heart.

Supplementary Table 4. Diagnostic performance of the modified Agüero method in blood EDTA samples from affected horses in Spain during the (1989-90) outbreak

Identification	Agüero 2008		Agüero-New-mod	
	Result	Ct	Result	Ct
1542/89 (44)	POS	20.3	POS	20.42
1542/89 (44)	POS	22.1	POS	22.72
1542/89 (43)	POS	20.4	POS	21.57
1542/89 (37)	POS	22.7	POS	23.62
1542/89 (33)	POS	22.9	POS	20.96
1542/89 (32)	POS	23.2	POS	22.91
1542/89 (28)	POS	24.8	POS	24.53
1542/90 (41)	POS	25.1	POS	24.94
1371/89 (51)	POS	31.1	POS	29.17
1371/89 (51)	POS	28.8	POS	28.01
1371/89 (5)	POS	30.2	POS	29.37
1371/89 (55)	POS	24.1	POS	24.06
1371/89 (6)	POS	30.6	POS	27.84
1371/89 (67)	POS	29.3	POS	27.57
1371/89 (8)	POS	28.5	POS	26.53
1371/89 (35)	POS	32.2	POS	31.13
1371/89 (28)	POS	33.7	POS	32.28
1371/89 (3)	POS	31.9	POS	30.88
1378/89 (41)	POS	23.6	POS	23.07
1378/89 (7)	POS	29.5	POS	29.61
1378/89 (71)	POS	31.1	POS	27.58
1378/89 (46)	POS	27.3	POS	25.61
1378/89 (32)	POS	31.4	POS	30.28
1378/89 (15)	POS	31.3	POS	28.23
	**Mean Ct**	27.34	**Mean Ct**	26.37
	**SD**	4.15	**SD**	3.46
	**Mean Ct absolute difference**			0.97
	***p*-value**			0.38

POS, positive sample; Ct, cycle threshold; SD, standard deviation

Supplementary table 5. Intra-assay repeatability of the modified-Agüero rRT-PCR method

AHSV prototype strain	Dilution	Rep1	Rep2	Absolute Ct value difference	AHSV prototype strain	Dilution	Rep1	Rep2	Absolute Ct value difference
AHSV-1	-2($10^{4.6}$)	20.17	19.06	1.11	AHSV-2	-2($10^{4.5}$)	20.43	21.41	0.98
	-3 ($10^{3.6}$)	23.13	23.73	0.6		-3 ($10^{3.5}$)	25.17	25.36	0.19
	-4 ($10^{2.6}$)	28.48	29.3	0.82		-4 ($10^{2.5}$)	27.97	28.91	0.94
	-5 ($10^{1.6}$)	34.77	31.12	3.65		-5 ($10^{1.5}$)	32.17	32.98	0.81
	-6 ($10^{0.6}$)	37.62	37.74	0.12		-6 ($10^{0.5}$)	35.82	36.93	1.11
	-7 ($10^{0.06}$)	37.1	neg			-7($10^{0.05}$)	36.92	neg	
	-8 ($10^{0.06}$)	neg	neg			-8($10^{0.005}$)	neg	neg	
AHSV-3	-2($10^{3.6}$)	22.86	21.96	0.9	AHSV-4	-2($10^{5.2}$)	22.21	21.64	0.57
	-3 ($10^{2.6}$)	26.17	26.19	0.02		-3 ($10^{4.2}$)	25.51	25.15	0.36
	-4 ($10^{1.6}$)	30.1	30.02	0.08		-4 ($10^{3.2}$)	29.74	28.61	1.13
	-5 ($10^{0.6}$)	32.89	33.14	0.25		-5 ($10^{2.2}$)	32.38	32.97	0.59
	-6 ($10^{0.06}$)	37.53	36.32	1.21		-6 ($10^{1.2}$)	36.38	36.95	0.57
	-7 ($10^{0.006}$)	neg	neg			-7 ($10^{0.2}$)	neg	neg	
	-8($10^{0.0006}$)	neg	neg			-8 ($10^{0.02}$)	neg	neg	
AHSV-5	-2($10^{3.9}$)	18.49	20.94	2.45	AHSV-6	-2($10^{5.1}$)	21.43	20.59	0.84
	-3 ($10^{2.9}$)	27.61	24.09	3.52		-3 ($10^{4.1}$)	25.52	24.5	1.02
	-4 ($10^{1.9}$)	28.07	28.04	0.03		-4 ($10^{3.1}$)	30.13	29.43	0.7
	-5 ($10^{0.9}$)	32.56	32.93	0.37		-5 ($10^{2.1}$)	33.13	33.4	0.27
	-6 ($10^{0.09}$)	37.32	37.02	0.3		-6 ($10^{1.1}$)	36.95	36.25	0.7
	-7 ($10^{0.009}$)	neg	neg			-7 ($10^{0.1}$)	neg	neg	
	-8 ($10^{0.0009}$)	neg	neg			-8 ($10^{0.01}$)	neg	neg	
AHSV-7	-2($10^{5.1}$)	26.07	24.79	1.28	AHSV-8	-2($10^{4.6}$)	23.9	23.75	0.15
	-3 ($10^{4.1}$)	28.16	28.09	0.07		-3 ($10^{3.6}$)	27.38	27.02	0.36
	-4 ($10^{3.1}$)	31.72	30.79	0.93		-4 ($10^{2.6}$)	32.18	31.92	0.26
	-5 ($10^{2.1}$)	36.48	35.09	1.39		-5 ($10^{1.6}$)	34.01	35.27	1.26
	-6 ($10^{1.1}$)	neg	38.62			-6 ($10^{0.6}$)	38.32	neg	
	-7 ($10^{0.1}$)	neg	neg			-7 ($10^{0.06}$)	neg	neg	
	-8 ($10^{0.01}$)	neg	neg			-8 ($10^{0.006}$)	neg	neg	
AHSV-9	-2($10^{5.1}$)	22.51	23.51	1					
	-3 ($10^{4.1}$)	26.35	25.67	0.68					
	-4 ($10^{3.1}$)	29.77	31.3	1.53					
	-5 ($10^{2.1}$)	32.07	34.89	2.82					
	-6 ($10^{1.1}$)	38.47	37.78	0.69					
	-7 ($10^{0.1}$)	neg	neg						
	-8($10^{0.01}$)	neg	neg						

Cuantitative differences between replicates

**Mean Ct difference**	**0.90**
**SD**	**0.84**

Rep., Technical replicate; Ct, cycle threshold; SD, standard deviation. Ct values obtained in each replicate and differences in absolute value between replicates are shown.
