# Supplementary material for: Modification and Validation of a Reference Real-Time RT-PCR Method for the Detection of a New African Horse Sickness Virus Variant
Source: Microorganisms. 2025 Nov 25;13(12):2684. doi: 10.3390/microorganisms13122684 (PMC12735099; doi:10.3390/microorganisms13122684)
Supplement: Supplementary file 1 [file microorganisms-13-02684-s001.zip › Sup_Fig_1.pdf]

Supplementary figure 1. Synthetic RNA sequences and features.

AHS SEGMENT-7 (VP7) target sequence of the RSA strain (AHSV-sRNA-RSA)

5'aauggguguuguugcgccaguaggccagaucaacagagcucuugugcuagc**g**gc**u**uaccacua
guggcugcgguguugcacgguc**g**ccgcuuucauuagugucgcgucggcucuuaugcug-3'

Base Count: 120
Purification method: HPLC
Molecular Weight (Da): 38.508
Tm(ºC): 91
OD_{260nm}: 1,2
nmol: 1,0
µl required for 100µM solution: 10

AHS SEGMENT-7 (VP7) target sequence of the AHSV reference strains (AHSV-sRNA-C+)

5'aauggguguuguugcgccaguaggccagaucaacagagcucuugugcuagc**a**gc**c**uaccacua
guggcugcgguguugcacgguc**a**ccgcuuucauuagugucgcgucgguucuuaugcug-3'

Base Count: 120
Purification method: HPLC
Molecular Weight (Da): 38.476
Tm(ºC): 90
OD_{260nm}: 1,2
nmol: 1,0
µl required for 100µM solution: 10

AHS, African Horse Sickness; VP7, viral protein 7; AHSV, African horse sickness virus; AHSV-RSA-sRNA, synthetic RNA containing the target sequence of the new South African variant (GenBank accession number PV455026) ; AHSV-sRNA-C+, synthetic RNA used as positive control containing the target sequence the AHSV reference strains. The complementary sequence of the primers and probe of the Agüero 2008 and modified-Agüero methods are highlighted in red, SNPs differing between the target sequence of the new variant and the target sequence of the reference strains are indicated in bold blue letters; HPLC, High performance liquid chromatography; Da, Dalton; Tm, melting temperature; OD, optic density.
