# Supplementary material for: Modification and Validation of a Reference Real-Time RT-PCR Method for the Detection of a New African Horse Sickness Virus Variant
Source: Microorganisms. 2025 Nov 25;13(12):2684. doi: 10.3390/microorganisms13122684 (PMC12735099; doi:10.3390/microorganisms13122684)
Supplement: Supplementary file 1 [file microorganisms-13-02684-s001.zip › Sup_Fig 2.pdf]

**Alignment of the segment 7 target sequences of the 9 AHSV serotypes reference strains.**

```
OL581620.1 (AHSV-1 29/62)     AATGAATGGTGTTGTCGCGCCAGTAGGCCAGATCAACAGAGCTCTTGTGC
KP939459.1 (AHSV-2 82/61)     GATGAATGGTGTTGTTGCGCCGGTAGGCCAGATCAACAGAGCTCTTGTGC
OL581640.1 (AHSV-3 13/63)     AATGAATGGTGTTGTCGCGCCAGTAGGCCAGATCAACAGAGCTCTTGTGC
OL581650.1 (AHSV-4 32/62)     AATGAATGGTGTTGTTGCGCCAGTAGGCCAGATCAACAGAGCTCTTGTGC
OL697715.1 (AHSV-5 30-62)     GATGAATGGTGTTGTTGCGCCGGTAGGCCAGATCAACAGAGCTCTTGTGC
gi|582057814|(AHSV-6 39/62)   AATGAATGGTGTTGTTGCGCCAGTAGGCCAGATCAACAGAGCTCTTGTGC
gi|582057816|(AHSV-7 62/31)   AATGAATGGTGTTGTTGCGCCAGTAGGCCAGATCAACAGAGCTCTTGTGC
gi|582057818|(AHSV-8 62/10)   AATGAATGGTGTTGTCGCGCCAGTAGGCCAGATCAACAGAGCTCTTGTGC
gi|582057823|(AHSV-9 90/61)   AATGAATGGTGTTGTCGCGCCAGTAGGCCAGATTAACAGAGCTCTTGTGC
                                  *********** *****.*********** ****************

OL581620.1 (AHSV-1 29/62)     TAGCAGCCTACCACTAGTGGCTGCGGTGTTGCACGGTCACCGCTTTCATT
KP939459.1 (AHSV-2 82/61)     TAGCAGCCTACCACTAGTGGTAGCGGTGTTGCACGGTCACCGCTTTCATT
OL581640.1 (AHSV-3 13/63)     TAGCAGCCTACCACTAGTGGCTGCGGTGTTGCACGGTCACCGCTTTCATT
OL581650.1 (AHSV-4 32/62)     TAGCAGCCTACCACTAGTGGCTGCGGTGTTGCACGGTCACCGCTTTCATT
OL697715.1 (AHSV-5 30-62)     TAGCAGCCTACCACTAGTGGTTGCGGTGTTGCACGGTCACCGCTTTCATT
gi|582057814|(AHSV-6 39/62)   TAGCAGCCTACCACTAGTGGCTGCGGTGTTGCACGGTCACCGCTTTCATT
gi|582057816|(AHSV-7 62/31)   TAGCAGCCTACCACTAGTGGCTGCGGTGTTGCACGGTCACCGCTTTCATT
gi|582057818|(AHSV-8 62/10)   TAGCAGCCTACCACTAGTGGCTGCGGTGTTGCACGGTCACCGCTTTCATT
gi|582057823|(AHSV-9 90/61)   TAGCAGCCTACCACTAGTGGCTGCGGTGTTGCACGGTCACCGCTTTCATT
                              ******************** :****************************

OL581620.1 (AHSV-1 29/62)     AGTGTCGCGTCGGTTCTTATGCTGATAAAGTACGCATAAGTAATACGTCA
KP939459.1 (AHSV-2 82/61)     AGTGTCGCGTCGGTTCTTATGCTGATAAAGTACGCATAAGTAATACGTCA
OL581640.1 (AHSV-3 13/63)     AGTGTCGCGTCGGTTCTTATGCTGATAAAGTACGCATAAGTAATACGTCA
OL581650.1 (AHSV-4 32/62)     AGTGTCGCGTCGGTTCTTATGCTGATAAAGTACGCATAAGTAATACGTCA
OL697715.1 (AHSV-5 30-62)     AGTGTCGCGTCGGTTCTTATGCTGATAAAGTACGCATAAGTAATACGTCA
gi|582057814|(AHSV-6 39/62)   AGTGTCGCGTCGGTTCTTATGCTGATAAAGTACGCATAAGTAATACGTCA
gi|582057816|(AHSV-7 62/31)   AGTGTCGCGTCGGTTCTTATGCTGATAAAGTACGCATAAGTAATACGTCA
gi|582057818|(AHSV-8 62/10)   AGTGTCGCGTCGGTTCTTATGCTGATAAAGTACGCATAAGTAATACGTCA
gi|582057823|(AHSV-9 90/61)   AGTGTCGCGTCGGTTCTTATGCTGATAAAGTACGCATAAGTAATACGTCA
                              ************************
```

Primers forward, complementary reverse and probe are represented by arrows and shown in red; mismatches of the primers/probe with the target sequences are highlighted in yellow; nucleotides of the Agüero 2008 probe and complementary reverse primer replaced by degenerate nucleotides in the modified method are shown in bold blue. Alignment performed with Clustal W (Larkin, M.A., et al. (2007) Clustal W and Clustal X Version 2.0. *Bioinformatics*, 23(21):2947-2948).
